# Symptom Tracking for Patient-Reported Outcomes in Cancer: User-Centered Design of the AthenaCompanion Web Application

**DOI:** 10.2196/94017

**Published:** 2026-08-05

**Authors:** Kyle Nolla, Laura M Perry, Anvitha Gogineni, Sheetal Kircher, Nisha Mohindra, Michael Bass, Macy K Tetrick, Gabriela Sanchez-Petitto, Anne Noonan, Carolyn Presley, David Cella, Roberto M Benzo

**Affiliations:** 1 Department of Psychology, Morgan State University, 1700 E Cold Spring Ln, Baltimore, MD, 21251, United States, 1 443-885-4215; 2 Department of Medical Social Sciences, Feinberg School of Medicine, Northwestern University, Chicago, IL, United States; 3 Center for Health Outcomes, Implementation, and Community-Engaged Science, Tulane University, New Orleans, LA, United States; 4 College of Medicine, Department of Internal Medicine, Division of Cancer Prevention and Control, Columbus, OH, United States; 5 Robert H Lurie Comprehensive Cancer Center, Northwestern Medicine, Chicago, IL, United States; 6 The Ohio State University Comprehensive Cancer Center, The Ohio State University Wexner Medical Center, Columbus, OH, United States; 7 Department of Internal Medicine, Division of Cancer Prevention and Control, College of Medicine, The Ohio State University, Columbus, OH, United States

**Keywords:** patient-reported outcomes, mobile health, gamification, user-centered design, symptom monitoring, older adults, cancer care

## Abstract

**Background:**

Routine monitoring of patient-reported outcomes (PROs) during cancer treatment improves symptom control and quality of life, yet real-world uptake and sustained engagement with PRO monitoring remain suboptimal. Gamification has been shown to improve engagement with digital health interventions. User-centered design approaches are needed to ensure that gamified PRO tools are acceptable, usable, and responsive to patient and clinician needs, especially for older adult users.

**Objective:**

This study aimed to develop a gamified symptom monitoring web application for older adult patients with cancer using a multiphase, iterative, and user-centered approach.

**Methods:**

The overall study design was a mixed-methods user-centered design study involving 3 phases. From 2022 to 2026, participants were recruited across online and clinical settings using multiple strategies, including ResearchMatch (Vanderbilt University Medical Center), clinician referrals, professional networks, and outreach through the electronic health record at an academic medical center. Phase 1 was a survey of older adults with chronic health conditions. They reported on symptom severity, mobile health (mHealth) preferences, and gamification preferences, which were analyzed descriptively. Phases 2 and 3 involved semistructured interviews with older adult cancer survivors and clinicians, respectively, to identify actionable feedback, followed by iterative usability testing with older adult cancer survivors.

**Results:**

In Phase 1, older adults (n=216) with chronic conditions reported high frequency of mobile phone use (across 11 mobile phone behaviors, mean 6.1, SD 1.63 where 6 indicates daily use) and generally favorable attitudes toward gamification (across 18 gameful design elements, means ranged from 2.8‐4.3 on a 1‐5 scale and SDs ranged from 0.75‐1.12), with learning elements rated most appealing (mean 4.3/5, SD 0.75). Concerns about a gamified mHealth app included data privacy and perceived trivialization of health. Phase 2 interviews (n=7) demonstrated strong interest in longitudinal symptom visualization and clinician-sharable reports; gamified content was viewed as engaging by most participants but was preferred as optional. In Phase 3, iterative clinician interviews (n=5) and patient usability testing (n=9) led to substantial refinements, including simplified navigation, enhanced visual accessibility, further guidance on score interpretation with embedded educational videos, and de-emphasis of the gamified travel learning component. Across usability testing rounds, the number of user experience problems per interview decreased from 17 to 4, indicating improved usability.

**Conclusions:**

Using a multiphase, mixed methods, user-centered design process, we developed AthenaCompanion (Northwestern University with The Ohio State University), a gamified web application for PRO monitoring tailored to older adults undergoing cancer treatment. Across surveys and interviews, patients emphasized the importance of clinical utility, clarity of symptom feedback, and low-pressure, optional gamification elements. This work demonstrates the feasibility of integrating gamification into PRO monitoring and provides a foundation for future work evaluating long-term usability, engagement, and clinical effectiveness in real-world oncology care.

## Introduction

Individuals undergoing cancer treatment often experience persistent physical and emotional distress that can interfere with daily life, impede treatment, and worsen clinical outcomes. To address patients’ symptom-related needs efficiently and effectively, many have called for a transformation within oncology care to routinely monitor symptoms through patient-reported outcome (PRO) assessments [1,2]. These calls are reinforced by an accumulation of clinical trials demonstrating that PRO monitoring can improve health-related quality of life, health care resource use, and in some cases, survival [3-6]. PRO monitoring interventions are most effective when assessments are completed frequently over several months during cancer treatment [3]. However, maintaining regular engagement with remote PRO monitoring can be a challenge for patients. For example, studies of system-wide implementation of PRO monitoring programs in oncology have found that only about 40%‐50% of eligible patients completed at least one PRO survey [7,8]. Furthermore, another study found that on average, patients complete only about 50% of the survey invitations they receive [9]. Therefore, developing and implementing strategies to improve patient uptake and maintenance of PRO monitoring should improve patient outcomes.

Although PRO use is multifactorial, one barrier is patients’ motivation to use (and continue using) available assessment platforms. Decades of research on behavior change theory emphasize the use of rewards to motivate individuals to maintain health behaviors [10], with autonomous motivations leading to long-lasting health behavior changes [11]. As technology has advanced, many mobile health (mHealth) interventions now target reward pathways by using “gamification” to incentivize specific behaviors. Past research suggests that health interventions using video game components (eg, personal avatars, points, badges, and leaderboards) facilitate participation and engagement [12,13]. Moreover, a recent scoping review found that gamified interventions for cancer self-management are growing and can increase adherence to clinically relevant health behaviors, such as medication taking, physical activity, and postchemotherapy fluid intake [13]. However, to our knowledge, there are no existing gamified interventions available that aim to increase patient adherence to PRO monitoring. Given that remote PRO monitoring interventions already rely on health information technologies, gamification is a logical strategy for enhancing user engagement.

The aim of this study was to conduct a multistage, mixed methods user-centered design process to design a gamified application for collecting PROs among individuals undergoing cancer treatment. Previous gamified interventions for cancer survivors have included multiple gamification elements, such as personal avatars, progress, levels, and aesthetically pleasing graphics and visuals inspired by video games [13]. However, there is a paucity of research highlighting older adult users’ preferences for gamified cancer and other health-focused apps [14]. Therefore, in Phase 1 of our multistage user-centered design process [15], we conducted an online survey of older adults with a chronic disease that asked about their technology use and comfort, preferences for various gamification elements, and associated background characteristics. Phase 2 engaged feedback from a user interface designer and patients, and Phase 3 engaged oncologists and patients to iteratively develop a gamified application for PRO monitoring.

## Methods

### Overview

**Figure 1.**
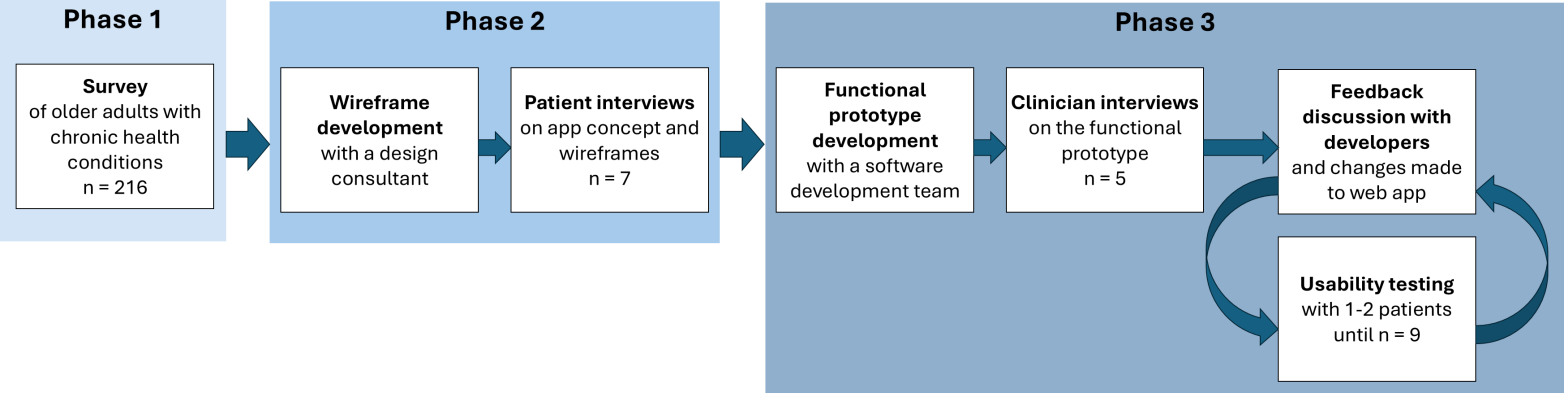
Diagram of the 3 phases of the study’s user-centered design process.

The design process was user-centered and iterative [16,17]. In short, we developed the web application with feedback from our intended users across multiple phases, with results from each phase informing decision-making in the next (see Figure 1). Phase 1 consisted of an online survey to assess the needs and preferences of intended users. Phase 2 included developing wireframes and collecting patient feedback on the application concept and wireframes. Phase 3 involved developing a functional prototype and refining it through clinician interviews and patient usability testing.

### Ethical Considerations

The Phase 1 survey was deemed exempt from review by the Northwestern University Institutional Review Board (IRB; STU00217420). Phase 2 patient interviews and Phase 3 clinician interviews were determined by the Northwestern University IRB to constitute product development activities and therefore not human subjects research (STU00218189). Phase 3 patient usability testing was approved as a multisite study by the Northwestern University IRB (STU00221487) and the Ohio State University IRB (2024X0127). Participant privacy and confidentiality were protected by separating identifying information from survey responses, interview transcripts, and other study materials. Identifying information was linked to study responses with an identification number, and the key file linking identifiers to the survey responses was kept in a secure REDCap (Vanderbilt University) database. All analyses were conducted on deidentified data only. Participants were compensated differently in each phase of the work. For Phase 1 surveys, participants were given the option to enter a raffle for one of twenty US $20 digital gift cards upon survey completion. For Phase 2 wireframe interviews, all participants received a US $100 digital gift card. For Phase 3 clinician interviews, all clinicians declined payment, although they were offered a US $40 digital gift card for participation. For Phase 3 patient usability testing, participants received a US $40 digital gift card for their time.

### Phase 1: Survey

This study was conducted and reported in accordance with applicable recommendations for digital health intervention development and web-based survey research (eg, the CHERRIES [Checklist for Reporting Results of Internet E-Surveys] [18]; see Multimedia Appendix 1).

#### Participants

We recruited a convenience sample through the online platform ResearchMatch (Vanderbilt University Medical Center), a national health volunteer registry supported by the US National Institutes of Health. Participants were eligible if they were aged 50 years, located in the United States, able to read English, and self-identified as having a chronic health condition. We intentionally sampled older adults with and without cancer to understand how symptom burden, self-management needs, and technology use patterns may differ according to cancer diagnosis. Full details of the survey features, design, and data handling are provided in Multimedia Appendix 1.

#### Measures

To describe overall health, we asked participants to report historical or current diagnoses of 22 common medical conditions. These 22 items were based on the US National Health Interview Survey [19] and the Functional Comorbidity Index [20], including an item for Long COVID.

To describe participants’ physical symptom burden, participants completed the Functional Assessment of Cancer Therapy-General (FACT-G) Physical Well-Being (PWB) subscale. In this measure, participants rate their experience in the last week of 7 common physical symptoms or concerns on a scale from 0 (Not at all) to 4 (Very much). Items are reverse-scored so higher scores indicate better physical well-being. Reliability was excellent (α=.90; *ω*=.91).

Participants reported their familiarity with technology using an abbreviated and adapted version of the Media and Technology Usage and Attitudes Scale (MTUAS) [21]. Participants report their frequency of completing certain activities (eg, read email on a mobile phone) on a scale from 1 (Never) to 10 (Always). We included 4 items from the original measure’s mobile phone subscale, modified an additional 4 phone-related items for relevance, and wrote 3 new items specific to our research interests in gamified mHealth (see Table S1A in Multimedia Appendix 1). From these 11 items, we selected 7 items relevant to desktop/laptop computers and asked about the frequency of these activities on the same scale (see Table S1B in Multimedia Appendix 1). The final 18-item adapted MTUAS had good-to-excellent reliability (α=.84; *ω*=.90).

To measure interest in specific gamification elements in an mHealth app, we drew from an existing published list of 59 gameful design elements [22]. We reviewed the list and excluded elements that were either incompatible with a Health Insurance Portability and Accountability Act (HIPAA)–compliant symptom-tracking app (eg, knowledge sharing, guilds, or teams which require user-to-user communication where protected health information [PHI] may be disclosed or inferred) or unfeasible given our time and budget (eg, creativity tools, narrative, or story, which require extensive creation of original material). The final list included 18 named elements and brief descriptions (eg, Learning: being invited to learn new skills that may be useful inside the system or in real life or Points: receiving points or experience for completing certain tasks; see Table S2 in Multimedia Appendix 1). As in the original study, participants rated each element on a 5-point scale from “Very unappealing” (1) to “Very appealing” (5). Reliability for this scale was excellent (α=.93; *ω*=.94).

Finally, participants were invited to respond to an open text prompt, “How do you feel about the idea of combining a video game with a health app?”

#### Planned Analyses

Because the survey’s purpose was to inform later design decisions, planned analyses were descriptive. First, we calculated means and SDs for each item of the adapted MTUAS and gameful design ratings and arranged them from high to low to understand the most frequent behaviors and preferences of users. Similarly, we calculated means and SDs for the number of comorbid diagnoses and the FACT-G scale to understand the average symptom burden of participants, then used a 2-tailed *t* test to investigate whether symptom burden differed by the presence or absence of a cancer diagnosis. Next, we used 2-tailed *t* tests to compare matched items within the adapted MTUAS that asked about mobile versus desktop platforms to understand which platform participants were more likely to use for digital symptom tracking. Finally, we used Welch *t* tests and ordinary least squares (OLS) regression to examine whether cancer diagnosis, sex, and age were associated with scores on adapted MTUAS items and gameful design preferences to assess whether design-relevant preferences were consistent across key demographics and clinical subgroups and, thus, appropriate to inform broadly applicable design decisions. To adjust for multiple comparisons, we applied the Bonferroni correction to all *P* values reported.

Open responses were analyzed using descriptive content analysis [23,24]. Two authors read through all responses several times, then separately created a list of emergent themes that were common across responses. Next, the coders together categorized each theme as reflecting a positive or negative attitude toward gamification in health apps. Finally, each participant was described as giving a positive, negative, or neutral (neither positive nor negative) response, if a response was given, such that the coders calculated an overall percentage of positive, negative, and neutral attitudes across participants.

### Phase 2: Wireframe Development and Patient Interviews

The next step in development involved the creation of a low-fidelity prototype with the help of a design consultant and soliciting patient feedback on these materials and the web application concept.

#### Wireframe Development

An independent user experience design consultant was hired to create a wireframe, or a visual representation of the web application’s key pages and flow. The consultant produced a needs statement, a list of guiding design principles, a style tile, and 12 concept images for specific application features. Next, a wireframe or an image-based presentation that showcased the web application’s intended functions and feel was assembled.

#### Patient Interviews

Two authors on the study (SK and NM) are clinicians at Northwestern Medicine and volunteered to conduct study recruitment by offering eligible patients a study information sheet. Participants were eligible for the study if they were aged 45 years with a documented cancer diagnosis. Physicians offered the study information sheet to eligible patients for a 2-week period. During that 2-week period, 7 participants (3 male and 4 female) were recruited. The average age of participants was 60 years; 5 were non-Hispanic White and 2 were non-Hispanic Black.

Interested patients contacted the researchers using the email address listed on the study information sheet. After confirming eligibility, 2 authors scheduled and conducted interviews (KN and LMP). Interviews were conducted over Zoom (Zoom Communications, Inc.) following a semistructured interview guide (see Multimedia Appendix 1). The interviewer made a copy of each interview guide per participant and took notes underneath each question as it was asked.

In the first part of the interview, participants were shown the app’s core feature, a “check-in” where they report their recent experiences with several symptoms using the patient-reported outcomes measurement information system (PROMIS) [25] family of computer-adaptive tests [26] as they learn about a travel destination of their choice. They were asked about their general impressions and opinions on specific planned features (travel facts, frequent flyer points, and collectible passport stamps). Next, participants were asked about the reporting and interpretation of their symptom scores. They were shown planned displays of immediate and longitudinal symptom scores and asked for opinions on the interpretability of graphs, desired support if scoring high, and more. Finally, participants were asked for general feedback on the application concept, including how they currently monitor symptoms, whether they or other patients with cancer would use the application, and areas for improvement. Interviews lasted approximately 30 minutes.

Interview notes were analyzed using rapid qualitative analysis with descriptive thematic coding [27,28]. In short, the interview guide served as a structured template for compiling the individual responses of all participants, then for summarizing across all responses. Two authors (KN and LMP) read through the summarized responses to each interview question to understand common needs and together used this information to inform design decisions.

### Phase 3: Prototype Development and Refinement

In the next phase, we approached several software development teams with our survey results, wireframes, and a summary of needs identified in patient interviews. We hired a development team based on quoted costs, timelines, and previous experience with mHealth web applications. Once development of a functional web application was complete, end users—both patients and clinicians—provided feedback that informed its refinement.

#### Clinician Interviews

Clinicians were eligible to participate if they were actively treating older adult patients with cancer at either Northwestern Medicine’s Lurie Cancer Center or The Ohio State Comprehensive Cancer Center. Researchers emailed eligible clinicians until at least 2 clinicians from each site agreed to participate. Two researchers scheduled the online interviews by email and conducted them via Zoom (KN and RMB).

Clinicians completed 30-minute semi-structured interviews on 2 topics (see Multimedia Appendix 1). First, they were shown the working prototype of the application, including the home page, symptom check-in process, gamified travel elements, symptom score graphs, and email-to-clinician feature, and asked for their thoughts. At this stage, they were also asked whether emails with attached reports were the most effective way for patients to communicate scores to them. Next, they were shown at least 2 pregenerated symptom score reports that are intended for clinician communication. They were asked to interpret the patient’s scores and for general feedback on what was effective or what was not about the graphs and tables displayed in the report.

As with Phase 2 wireframe interviews, interviewers printed a copy of each interview guide and kept notes underneath each question as it was asked. Interviews were again analyzed with a rapid qualitative approach, where each response was compiled under the appropriate question, the researchers generated a summary for each question, and the summaries were read through by the 2 team members to inform design decisions [27,28].

#### Usability Testing With Patients

Participants were recruited by email invitation. Email addresses were collected from patients at Northwestern Memorial Hospital who had agreed to be contacted for research studies. We retrieved a list of eligible participants from the Northwestern Medicine Enterprise Data Warehouse and contacted them in small groups until we reached our intended sample size of 8-10 participants. Interviews were conducted by 2 researchers at a time (KN, RMB, and AG). Participants were eligible if they were aged 50 years, understood English, and had a cancer diagnosis.

For usability testing, we prepared a list of key tasks within the application, such as navigating to the home screen, viewing past score reports, downloading a score report, and similar tasks (Multimedia Appendix 1). Participants were asked to complete each task without the experimenter’s help and to think aloud as they did so. Each usability testing session lasted about one hour. Participants provided informed consent to record the interviews, which were transcribed by a university-approved AI transcription service for analysis.

We coded interviews for user experience problem instances (UPIs) in which users performed the task incorrectly, did not know what to do next, or expressed frustration, and summarized these as user experience design problems (UDPs), an established method in user experience research [29,30]. Coding was conducted by 2 researchers (AG and RMB). We conducted usability testing in rounds of one to 2 participants, continuing until sufficient UPIs were identified to justify another development sprint. Because multiple usability issues could often stem from a single UDP, the research team synthesized findings after each round to define design challenges rather than treating issues in isolation.

For each UDP, the research team ideated potential design solutions and presented these to the development team to assess technical feasibility and required hours of effort and to solicit alternative implementation approaches. Based on this feasibility input and budgetary constraints, the team collaboratively decided which solutions to implement. After these solutions were implemented, we resumed usability testing with the next 1-2 participants. This iterative cycle continued until funds were exhausted, after a total of 9 usability interviews.

## Results

### Phase 1: Survey

#### Participant Characteristics

The anonymous survey was begun by 378 people. We excluded 124 responses that did not complete the survey and 38 participants who missed 2 or more attention check questions for a final sample of 216 participants.

Demographic data included age, sex, race, ethnicity, education, insurance coverage, and perceived financial well-being [31], as reported in Table 1. Participants could select multiple categories for race and insurance.

Participant-reported sex was 72% (n=155) female and 27% (n=59) male. For ethnicity, 7% (n=15) of participants self-described as Hispanic/Latino, and 93% (n=201) self-described as not Hispanic/Latino. For race, participants self-described as White (n=152, 70%), Black (n=47, 22%), Native American/Pacific Islander (n=12, 5%), and Asian (n=8, 4%); 7% (n=15) selected more than one race. Around 72% of the sample had a Bachelor’s degree or higher education level. Around 78% described their financial well-being as “very comfortable” or “doing okay,” slightly higher than the 2022 national average of 72% of Americans in these categories. Participants reported a mean of 3.1 (SD 2.12) past or current diagnoses from the list of 22 common diagnoses given. A total of 32 (15%) participants indicated having a current or past cancer diagnosis. In terms of symptom burden, the average score for the FACT-G PWB scale was 20.1 (SD 6.00), which indicates significantly lower function than the general population mean of 22.7 (SD 5.4) [32] in a 1-sample *t* test (*t*_215_=–6.3; *P*<.001). Patients with a prior or current cancer diagnosis did not significantly differ in their FACT-G PWB scores from other participants (*F*_1, 214_=5.99; *P*=.76).

**Table 1.** Study 1 participant characteristics.

Characteristic (N=216)	Statistic
Age, mean (SD)	64.3 (8.9)
Sex, n (%)	
Female	155 (72)
Male	59 (27)
Not reported	2 (1)
Race and ethnicity, n (%)	
Non-Hispanic White	138 (64)
Non-Hispanic Black	46 (21)
Hispanic/Latino	15 (7)
Others	17 (8)
Education, n (%)	
High school diploma or equivalent	6 (3)
Some college	21 (10)
Certificate or technical degree	7 (3)
Associate degree	25 (12)
Bachelor’s degree	71 (33)
Master’s degree	58 (27)
Doctoral degree	26 (12)
Insurance, n (%)	
Medicare	119 (55)
Private insurance	111 (51)
Medicaid	19 (9)
No insurance	3 (1)
Financial well-being, n (%)	
Living comfortably	84 (39)
Doing okay	84 (39)
Just getting by	31 (14)
Finding it difficult to get by	15 (7)
No response	2 (1)
Clinical characteristics	
Number of prior or current diagnoses, mean (SD)	3.1 (2.12)
Prior or current cancer diagnosis, n (%)	32 (15)
FACT-G PWB^a^	20.1 (6.00)

a FACT-G PWB: Functional Assessment of Cancer Therapy-General Physical Well-Being.

#### Familiarity With Technology

Participants used their mobile phones extremely frequently; we took the maximum frequency of use across all phone items, and the median for this value was 9 (several times per hour). Across all 11 mobile phone–related behaviors, the mean value was 6.1 (SD 1.63), where 6 indicates daily use. In contrast, participants used computers daily but not as frequently as phones: the median for maximum computer use was 7 (several times per day). Across all 7 computer-related behaviors, the mean value was 5.4 (SD 2.06), where 5 is several times per week. To summarize trends, participants reported daily or greater engagement for the majority of mobile phone activities but weekly to daily engagement with browsing the web for health information, using apps for health-related activities, playing games, and sending emails. These results informed design decisions in Phases 2 and 3 (Table 2). Item-level results, including means and SDs for each item, are described in Table S1A in Multimedia Appendix 1.

For all 18 items, mean scores did not differ between participants with and without cancer (all *P*>.10). Means differed by sex for 2 items: women were more likely to play games using a mobile phone (*t*_105.2_=3.41; *P*=.01), while men were more likely to use a computer to browse the web for any purpose (*t*_110.5_=−3.26; *P*=.01). Finally, in individual linear regression, greater age predicted lower frequency of 4 technology-related behaviors: sending and receiving text messages (*t*_213_=−3.87; *P*=.002; *R*^2^=.07), reading emails on a phone (*t*_212_=-3.68; *P*=.006; *R*^2^=.06), browsing the web on a phone (*t*_212_=−4.22; *P*<.001; *R*^2^=.08), using mobile apps for any purpose (*t*_213_=−3.32; *P*=.02; *R*^2^=.05), and using health-related mobile apps (*t*_213_=−3.32; *P*=.02; *R*^2^=.04). These tests are also described for each item in Table S1A in Multimedia Appendix 1.

Comparing matched items for mobile phone versus computer use, only 2 items showed statistically significant differences after correcting for multiple comparisons: participants were more likely to use mobile phones than computers during nonworking hours (*t*_211_=8.48; *P*<.001) and more likely to play games on phones than on computers (*t*_211_=5.89; *P*<.001; see Table S1B in Multimedia Appendix 1).

**Table 2.** How Phase 1 results informed Phases 2 and 3 design decisions.

Result	Design decision
Phones vs computers: much more frequent use of phones than computers, especially during nonworking hours and for games.	Optimize the web application and user-facing materials for mobile phones rather than computers.
Frequency of specific activities: health-related and game-related features were used less than daily but at least weekly on mobile phones.	Design the application and gamification features to reward weekly, rather than daily, engagement.
Gamification elements: learning was a clear favorite, and most other common gamification elements were rated favorably.	Incorporate learning into the application and include other favorably rated gamification elements as feasible.
Descriptive content analysis: a slight majority of users (55%) responded positively to the idea of a gamified mHealth^a^ app for symptom tracking, so interest is not universal. Negative opinions focusing on data security were noted.	Focus on the app’s function for symptom tracking first and foremost: gamification is not the selling point. Ensure information about data security and privacy is easily accessible.
Age: several key phone-related behaviors such as texting, emailing, and using health-related apps decreased in frequency with age, and certain gamification elements were less popular with older users.	Focus on our target age range of users aged 50‐70 years; we assumed familiarity with basic technology functions such as texting and emailing, but not more advanced functions such as downloading and locating a PDF on a smartphone.
Cancer diagnosis: patients with cancer or a cancer history did not differ meaningfully from patients with other chronic conditions in technology-related behaviors and preferences.	This finding supports the generalizability of our survey results to a cancer-focused symptom monitoring application due to a comparable level of familiarity and engagement across diagnostic groups.
Sample: our sample was nonrepresentative of cancer patients in sex, race/ethnicity, and socioeconomic status.	Prioritize recruitment of diverse patients in further interviews for application development to ensure broad appeal.

a mHealth: mobile health.

#### Preferred Gamification Elements

Across all 18 elements, mean ratings ranged from 2.8 to 4.3 on a 1‐5 scale, with SDs ranging from 0.75 to 1.12 (see Table S2 in Multimedia Appendix 1). A total of 16 elements had mean scores between Appealing (4) and Very Appealing (5). Learning, or being invited to learn new skills that may be useful inside the system or in real life, was the only element with a mean rating above 4 (mean 4.3, SD 0.75).

Using Welch *t* tests and regression analyses with Bonferroni correction, we found no differences in ratings based on cancer diagnosis or sex. Two elements were rated lower as participant age increased: points (*t*_212_=−3.6; *P*=.007; *R*^2^=.06) and avatars (*t*_214_=−3.2; *P*=.03; *R*^2^=.05).

#### Descriptive Content Analysis

Participants (n=209) responded to the open response prompt, “How do you feel about the idea of combining a video game with a health app?” Of these, 114 (55%) had positive opinions, 48 (23%) had negative opinions, and 40 (19%) had neutral opinions. Positive opinions commonly cited gamification as a way to “make a chore fun,” “good for the brain,” or as previously successful in other apps, such as Duolingo (Duolingo, Inc; a popular gamified app for language learning). Themes of negative opinions included rejection of all games in any form (“I’m not a gamer, so no”), fear that gamifying a health app trivializes its importance (“My health is not a game”), and concerns about data privacy and safety. Neutral opinions were defined by a lack of opinion (“Don’t know, don’t care”) or conditional interest (“Maybe but it depends on the way it’s done”).

### Phase 2: Patient Interviews

#### Wireframe Development

Together with our consultant, we identified the web application’s target user as a middle-aged to older adult managing cancer symptoms who has a baseline familiarity with technology and needs a nondisruptive tool to monitor and manage their health. The design principles were that the application should feel professional and medical, validate and inform the user, provide a sense of ease, and be pleasant and rewarding. The application should avoid mental burden for users or overly gendered, childish, or abstract features and presentation. Throughout these discussions, world travel was identified as a topic of interest for learning.

**Figure 2.**
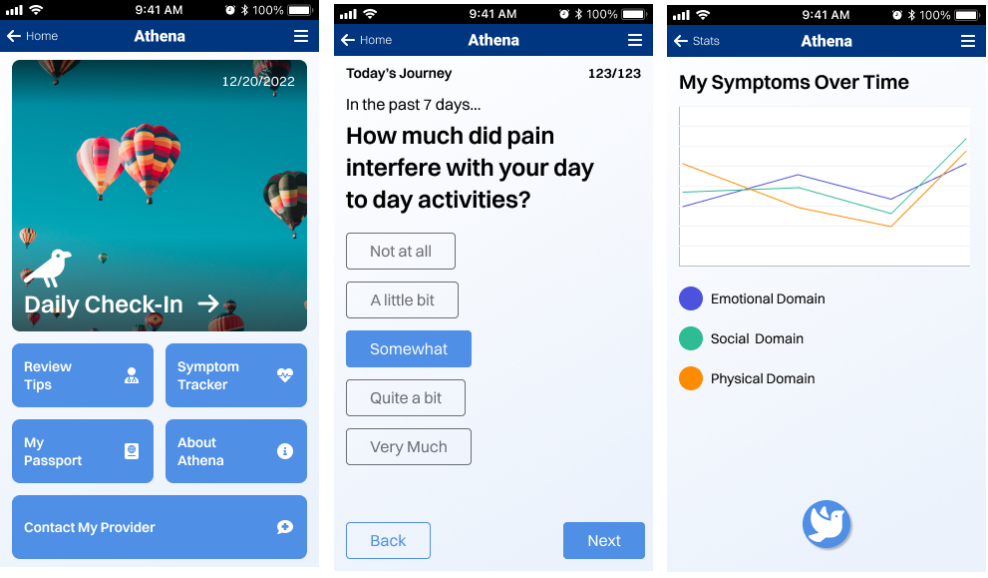
Examples of mock-ups used within the wireframe.

The consultant created several mock-ups of pages for key features of the application, presented in Figure 2. These mock-ups were arranged into a wireframe to demonstrate the flow of a symptom check-in for users. The wireframe depicted symptom reporting interwoven with travel facts as small rewards for completing each symptom.

#### Patient Interviews

We identified several key themes related to participants’ preferences (see Table S3 in Multimedia Appendix 1). All participants expressed strong interest in symptom tracking and longitudinal visualization, identifying longitudinal symptom score graphs as the most useful feature. Participants also reported a preference for supportive features following a high symptom score, such as educational guidance and options to contact their providers.

Reactions to gamification features were generally positive. However, some participants found the gamified travel elements distracting or unnecessary and suggested they be made optional. Competitive elements such as streaks or deadlines were viewed negatively. Most participants indicated that they would use the application if it were available publicly.

Based on this feedback, we made several key design decisions based on the wireframe interviews, described in Table 3 below.

**Table 3.** Actionable results from patient interviews on concept and wireframe.

Result	How it informed development
Symptom monitoring functions	
Strong interest in data visualization of symptom severity over time.	Heavily emphasized data visualization features that communicate score severity and change over time.
Desire for in-app and clinician-based support for severe symptom scores.	Implemented a downloadable symptom report that could be shared with clinicians to facilitate follow-up. In addition, we incorporated a structured response feature allowing interventionists to provide links to resources aligned with participants’ reported symptom severity, enabling timely and personalized symptom self-management support.
Desire to add a note to a check-in to provide context (eg, “received treatment” or “visited family today”).	Added a customizable free-text entry field with each symptom check-in, allowing participants to provide contextual details that enhance the clinical usefulness of their report.
Suggestion for push notification or email reminders.	Created weekly opt-in email and SMS text message reminders.
Gamified features	
Generally but not wholly positive reception to travel theme.	Incorporated travel into the regular check-in flow by default, with the ability for users to turn it off in settings.
Certain gamification mechanics (passport stamps and badges) preferred over others (points and streaks).	Incorporated a passport stamp collection mechanic with a progress bar on the home page to encourage continued engagement.

### Phase 3

The development process involved 2-week “sprints,” which allowed a clear structure for assigning tasks and giving feedback as work progressed. The development team provided a written timeline to guide expectations.

The first functional version of the web application was created in 4 sprints, and further iterative changes were made based on time estimates for the specific changes requested.

Core features included (1) integration of the PROMIS API for in-app administration of computer adaptive testing (CAT) [33]; (2) a symptom-reporting “check-in” flow involving reporting of multiple symptoms interspersed with travel destination content; (3) patient-facing pages explaining symptom scores and changes in scores over time; and (4) a clinician-facing summary report of symptom scores over time.

As a standard part of development, the development team checked that each page of the web application met accessibility standards (ie, Web Content Accessibility Guidelines, WCAG 2.1) for labeling, contrast, and function on screen readers and other assistive technologies. Using the WAVE (Web Accessibility Evaluation Tool; Web Accessibility In Mind [WebAIM]) scoring system, all pages of the final prototype had accessibility impact (AIM) scores between 8.7 and 9.8, indicating good-to-excellent accessibility.

#### Clinician Interviews

Two clinicians were recruited from Northwestern Medicine, which uses PROs as part of routine care. Three clinicians were recruited from The Ohio State University Wexner Medical Center, which has not implemented PROs in routine care. Clinicians focused on a wide range of cancer types, including gastrointestinal, thoracic, and hematological. Clinicians pointed out several practical implications for clinical use of the application. Specifically, themes included electronic health record (EHR) integration, management of patient expectations, and patient accessibility. They also gave fine-grained feedback on the clinician-facing report generated by the application to improve the speed and ease of clinical interpretation, especially for clinicians who had not previously worked with PRO data. A summary of issues and implemented solutions is provided in Table 4.

**Table 4.** Summary of clinician feedback gathered in interviews and solutions implemented.

Clinician feedback	Solution
Overall feedback	
AthenaCompanion (Northwestern University with The Ohio State University) symptom reports for clinicians should be uploaded to the EHR^a^ or presented in person, not sent to clinicians via email.	Remove feature to email reports and replace with a PDF download for patient users.
Patient expectations about their doctor’s access to the app’s content should be managed proactively.	Instruct patients on how to present their symptom reports for discussion with their provider to the report PDF and as a daily tip within the application.
Older adult patients may need additional visual support to interact with the application.	Increase the size of font, buttons, and lines on graphs. Added numerical labels to the progress bar displayed during check-ins.
Clinician-facing report feedback	
Create easily identified visual indicators for significant score changes.	Added a color-coded column to the report indicating whether a symptom had improved or worsened across the last 30 days.
Create visual indicators for patient-flagged events that may impact symptom scores.	Added red flag icons to days in which patients left a note during their check-in.
Visually separate symptom scores for which higher numbers mean better health and higher numbers mean worse health.	Divided these types of symptoms onto a separate page with a thick line on the graph indicating that scores above/below the line are moderate-to-severe.

a EHR: electronic health record.

#### Usability Testing With Patients

Nine participants completed interviews. The average age was 59 years, with 5 female and 4 male participants recruited. Six participants were non-Hispanic White, 2 were non-Hispanic Black, and one was Hispanic/Latino.

We analyzed design problems and created corresponding solutions after each interview. Solutions were implemented by the website developers after every 1 or 2 interviews. We organized our proposed solutions by order of potential impact, then presented the list to the developers for discussion. Solutions were selected for implementation based on impact, complexity, and budget constraints.

**Figure 3.**
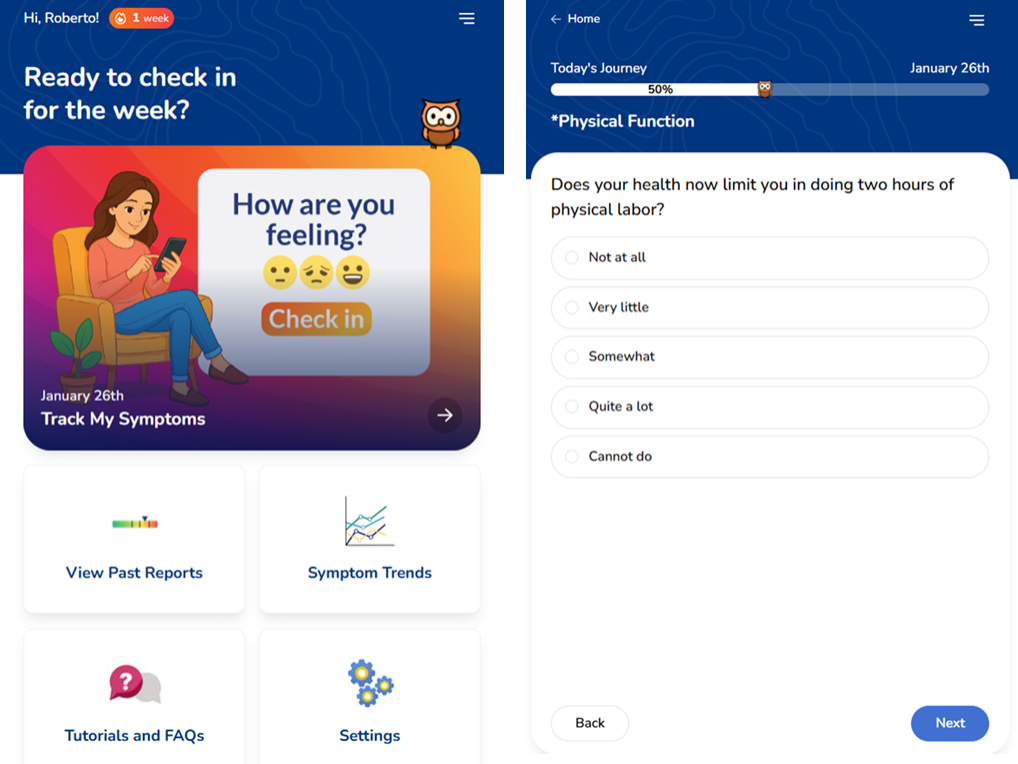
Finalized prototype after iterative usability testing: screenshots of the home screen featuring a customizable main tile that leads to the application’s main feature of symptom reporting (left) and symptom check-in screen featuring an item from the patient-reported outcomes measurement information system (PROMIS) physical function (right).

**Figure 4.**
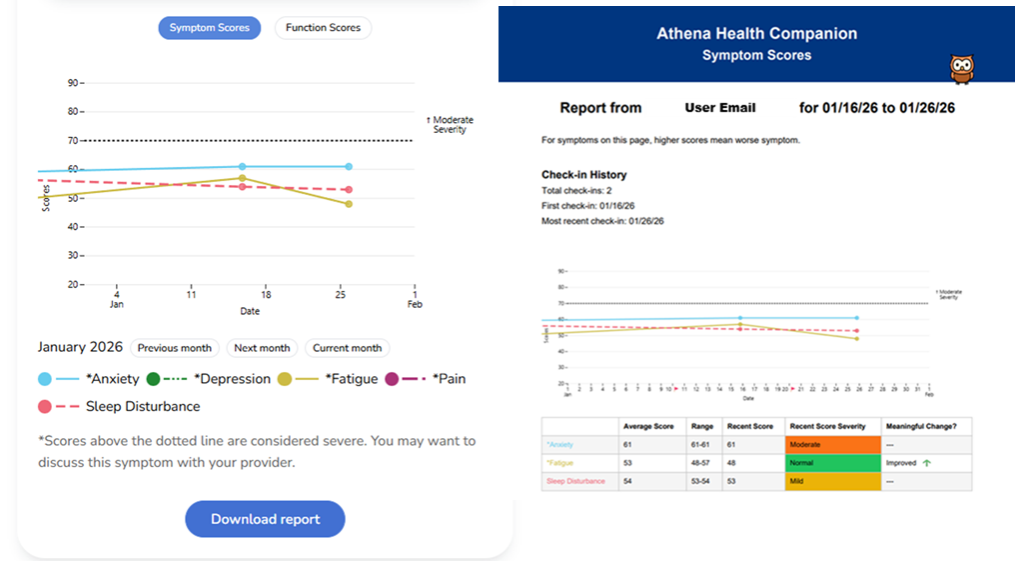
Finalized prototype after iterative usability testing: screenshots of display of longitudinal symptom changes for both patients (left) and in the clinician-facing application report (right).

**Figure 5.**
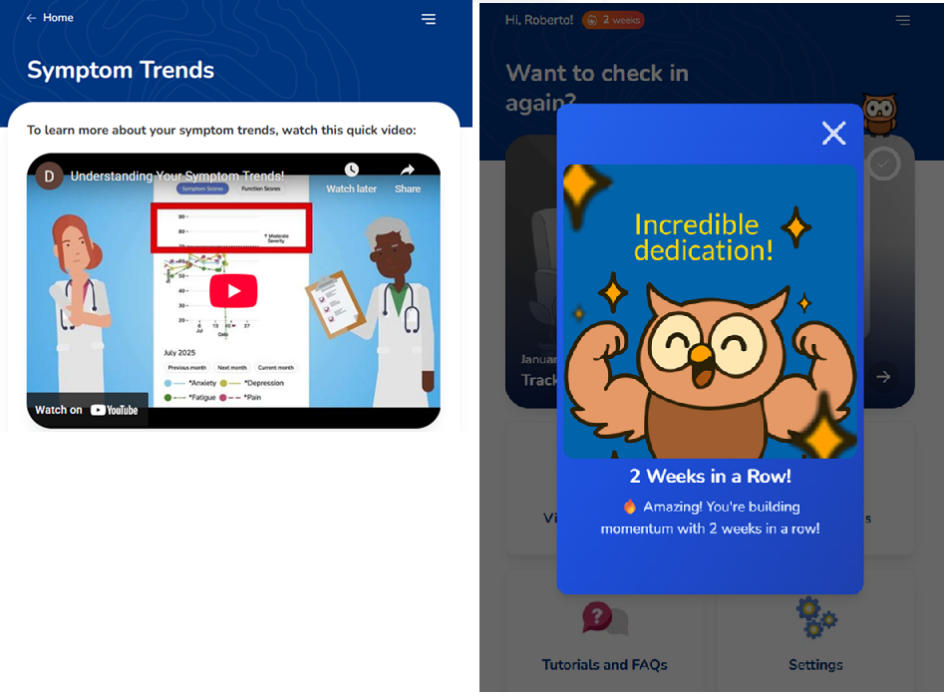
Finalized prototype after iterative usability testing: screenshots of 2 new features emerging from the iterative development process: an embedded video guide on interpreting symptom trends (left) and encouraging messages when weekly streaks are achieved (right).

For the first 3 interviews, usability testing with think-aloud protocol revealed an average of 17 problems and 12 corresponding solutions each. In the final 3 interviews, participants had an average of 4 problems with 3 corresponding solutions. Major changes are summarized in Table 5. Screenshots of the completed version of the application are presented in Figures 3–5.

**Table 5.** Summary of major application changes that were informed by patient usability testing.

Patient feedback	Solution
The multistage sign-up process resulted in several pain points	Streamlined the sign-up process to be via a single email link
Difficulty navigating to or from key application locations (eg, home page, score trends over time, and settings page)	Simplified the home screen interface; increased the default font size and relative size of navigation buttons
Difficulty understanding PROMIS^a^ score meanings (eg, *t* score metric) without researcher explanation	Created an animated video, “Learning about Your Symptom Report” explaining score interpretation
Difficulty knowing exact symptom scores when viewing the longitudinal score report interface	Created an animated video, “Understanding Your Symptom Trends” to give step-by-step instructions
Confusion when prompted to select travel destinations at the start of symptom reporting; confusion seeing travel destinations throughout symptom reporting	Prompt participants at the start of symptom reporting to engage with travel content or not
Due to reduced emphasis on the travel concept, needed another rewarding gamified activity	Added “streaks” feature

a PROMIS: patient-reported outcomes measurement information system.

The single most effective change was the creation of patient-facing videos. Because patients struggled to interpret PRO scores and trends over time without researcher explanation, we recorded several 2-3 minute videos and embedded them at the top of each page where they were relevant (see Figure 4). After implementing videos between the third and fourth interviews, subsequent participants were able to interpret their PRO scores accurately and independently.

The most significant change to the app’s original design was the de-emphasis of the gamified travel feature. While the travel feature was not universally disliked, it did create confusion at key points during the symptom reporting process; thus, supporting our decision to design an alternative option. Thus, we changed the flow of symptom reporting; after clicking the large home icon to initiate symptom reporting, participants were presented with the option to engage with travel content rather than being presented with travel content by default (see Figure 4). Participant feedback on this change was positive.

Given that the travel feature was a key site of gamification, with patients rewarded for learning with passport stamps, we decided to test an additional gamification mechanic to ensure the application still provided gamified rewards. Recent work suggested that activity streaks, which reward consistent engagement over time (eg, 10 days in a row), are particularly effective for behavioral change [34]. Because patient feedback in the early stages of design suggested that streaks were undesirable due to perceived pressure, we designed the streak feature to be as low-pressure as possible; logging in once per week resulted in a new graphic of the application’s owl avatar celebrating the streak (see Figure 4), and breaks in the streak were not punished or even acknowledged except that the streak counter restarted. This change was implemented late in the design cycle; thus, we received feedback from only 2 participants, which is insufficient for evaluating its appropriateness and effectiveness. The streak feature is being more thoroughly evaluated in an ongoing multiweek pilot test of the application, where we will analyze engagement patterns before and after streak breaks, and directly ask about streaks in postintervention interviews.

## Discussion

### Principal Findings

This study applied an iterative, mixed methods, user-centered design process to develop AthenaCompanion (Northwestern University with The Ohio State University), a gamified mHealth web application for PRO monitoring among individuals undergoing cancer treatment. Across 3 phases, we identified 3 main findings. First, older adult users undergoing cancer treatment had a strong interest in symptom tracking with longitudinal visualization. Second, most were open to supportive, low-burden gamification elements such as learning and progress-tracking; a minority had a strong negative reaction to the idea of gamification. Finally, both patient and clinician users had high expectations for usability and functionality, with a desire for features that improve patient-provider communication such as EHR integration and direct reporting of symptoms to providers. These findings informed the design of AthenaCompanion, including its emphasis on data visualization and optional gamified features.

This study informs literature on PRO adherence challenges in real-world clinical contexts. Participants consistently identified longitudinal symptom visualization as one of the most valuable features of the application, as seen in some previous work [35,36], suggesting that clear, interpretable, and clinically relevant feedback may improve patient adherence in other PRO monitoring contexts [37]. Results from our clinician and patient interviews suggest that EHR integration of symptom trends over time can reduce burden for both clinicians (eg, by centralizing score reports) and patients (eg, by avoiding the need to locate and present score reports during appointments). Indeed, EHR-integrated PRO dashboards have successfully improved shared decision-making and reduced patient anxiety in some clinical contexts [38,39]. Accordingly, longitudinal symptom data displays should be considered essential for PRO monitoring applications both within and outside of the EHR. These visualizations may aid in shared decision-making and obtaining patient buy-in for continued symptom monitoring.

Clinician interviews and usability testing demonstrated that both patients and clinicians require support in interpreting PRO data, underscoring the need for intuitive visualizations such as bar charts, line charts, icons, and color-coding to aid discussion [40-42]. Given that clinicians vary in their familiarity with interpreting PROs, these design considerations are especially important for developers of patient-driven apps such as AthenaCompanion. Taken together, our findings suggest that patient PRO adherence may be improved by designing user-friendly systems that actively support interpretation, communication, and shared decision-making between patients and clinicians.

This study also informs the literature on gamification in mHealth contexts, especially for older adult users. Overall, older adults rated low-pressure, supportive elements such as learning and badges above competitive elements like leaderboards and streaks. These findings suggest that gamification may support engagement in this population, but gamification mechanisms that support autonomous, internal motivation should be prioritized above quick external rewards [11]. This is in line with previous results showing small benefits of gamification in older adult users [14].

As a whole, these findings highlight the importance of aligning digital health design with both behavioral science principles and patient-lived experiences. While gamification is often conceptualized in terms of external rewards, our results suggest that its effectiveness for patients with cancer depends on its ability to support patients’ intrinsic motivation and autonomy. Participants consistently emphasized that their engagement depended on the application’s clinical relevance and ability to provide clear feedback in the context of symptom burden and treatment demands. Indeed, in real-world PRO contexts, high symptom burden has been associated with lower digital PRO adherence [43,44]. Effective technology design for older adults with cancer must go beyond general principles of engagement—such as the assumption that gamification will broadly increase engagement, or that frequent engagement is ideal—or even general principles of design for older adults, such as simplicity, accessibility, and low cognitive burden, to incorporate the unique demands of cancer care. The user-centered design process allowed us to incorporate our users’ experiences of symptom burden and frequent ongoing care into design decisions that prioritize non-intrusiveness, interpretability, and clinical relevance.

### Limitations

The study has several important limitations. First, all phases of the study relied on selected samples. Phase 1 survey respondents were predominantly female, non-Hispanic White, and had advanced educational degrees; these demographic characteristics are associated with higher health literacy, comfort with digital technologies, and engagement with self-management tools; thus, our survey results may not reflect the experiences or preferences of more diverse populations. Similarly, Phase 2 and 3 interviews relied on convenience samples drawn from academic hospital systems, limiting generalizability to other care settings.

In addition to sampling limitations, the application has several constraints that were not able to be addressed within the timeline and budget of this development study. For example, symptom reports are currently exported to a PDF, which then must be manually added to the patient’s EHR; this process will limit scalability and adoption in real-world settings. The app’s current architecture includes structured data storage compatible with future Health Level Seven Fast Healthcare Interoperability Resources (HL7 FHIR) integration; thus, we hope to prioritize seamless EHR integration in future development cycles. Second, the late addition of the streaks feature was not fully evaluated during this study and may need refinement in future development. Finally, the cartoon owl mascot was well-received in individual interviews but may be unappealing to users who worry about trivialization of health as identified in Phase 1; the mascot may need redesigning depending on its reception in a broader sample.

The final limitation of note is that the application was developed and tested in brief interviews, without extended real-world use. Opinions expressed during the interviews may not accurately reflect real-world use during active cancer treatment, when fatigue and symptom burden most impact engagement, or in real appointments, when clinicians have limited time to interpret symptom reports. A prospective pilot study is currently underway to evaluate real-world engagement and acceptability. Until further testing is complete, the impact of AthenaCompanion on clinical outcomes, PRO monitoring adherence, or patient-provider communication remains unknown.

### Conclusions

This study demonstrates the feasibility of applying user-centered design approaches to the development of mHealth web applications that support symptom monitoring in older adults with cancer. By identifying patient and clinician preferences using a mixed methods and iterative approach, AthenaCompanion achieved a functional prototype with acceptable usability and clinical relevance. However, broader implementation is necessary to evaluate accessibility across diverse populations and real-world effectiveness. Future research should focus on longitudinal evaluation of a more diverse patient population in a real-world setting.

More broadly, this work highlights the importance of aligning digital health design with both behavioral science principles and patients’ lived experiences. Our findings suggest that successful gamification in oncology contexts may depend less on traditional reward-based gamification mechanics and more on engagement strategies that match patients’ clinical needs, emotional states, and desire for meaningful and actionable feedback.

## Supplementary material

10.2196/94017 Multimedia Appendix 1 The multimedia appendix includes recruitment text, the Checklist for Reporting Results of Internet Surveys (CHERRIES), the modified Media and Technology Usage and Attitudes scale, descriptive statistics and *t* test results for individual items in Phase 1, interview guides for Phases 2 and 3, and themes from Phase 2 patient interviews.

## Abbreviations

AIM: accessibility impact
CAT: computer adaptive testing
CHERRIES: Checklist for Reporting Results of Internet E-Surveys
EHR: electronic health record
FACT-G: Functional Assessment of Cancer Therapy-General
FACT-G PWB: Functional Assessment of Cancer Therapy-General Physical Well-Being
HIPAA: Health Insurance Portability and Accountability Act
HL7 FHIR: Health Level Seven Fast Healthcare Interoperability Resources
IRB: Institutional review board
mHealth: mobile health
MTUAS: Media and Technology Usage and Attitudes Scale
OLS: ordinary least square
PHI: protected health information
PRO: patient-reported outcome
PROMIS: patient-reported outcomes measurement information system
UDP: user experience design problem
UPI: user experience problem instance
WAVE: Web Accessibility Evaluation Tool
WCAG: Web Content Accessibility Guidelines
WebAIM: Web Accessibility In Mind
