# Supplementary material for: Symptom Tracking for Patient-Reported Outcomes in Cancer: User-Centered Design of the AthenaCompanion Web Application
Source: J Med Internet Res. 2026 Aug 5;28:e94017. doi: 10.2196/94017 (PMC13441117; doi:10.2196/94017)
Supplement: Supplementary file 1 [file jmir-v28-e94017-s001.docx]

**Supplementary Materials**

**S1.** ResearchMatch recruitment text.

**S2.** Checklist for reporting results of internet surveys (CHERRIES) for Phase 1.

**Table S1A.** Modified version of the Media and Technology Usage and Attitude scale used in Phase 1.

**Table S1B.** Comparing matched items for mobile phone versus computers from Phase 1.

**Table S2.** Abbreviated list of gameful design elements used in Phase 1.

**S3.** Phase 2 wireframe interview guide.

**S4.** Phase 3 clinician interview guide.

**S5.** Phase 3 patient usability test guide.

**Table S3.** Themes from Phase 2 patient interviews.

**S1.** ResearchMatch recruitment text.

Are you a U.S. resident over the age of 50 with a diagnosis of a chronic health condition?

If so, you may be qualified to participate in an online survey about technology use and game design preferences. The study is a one-time anonymous survey that takes about 30 minutes to complete. As a participant, you will be asked to answer survey questions about your demographics, health status, technology use, and preferences on different game design elements for a hypothetical web-based health app. We hope that the study will build knowledge that can help individuals with chronic conditions to be more satisfied with their care.

If you are interested in participating, please select the option “Yes, I’m interested” and proceed to the survey that follows. The first page of the survey will provide additional details about the study that you can read before deciding to participate. It will also provide contact details for the research team. We would be happy to answer any questions you may have before you decide to participate.

Laura M. Perry, PhD

Kyle Nolla, PhD

Northwestern University

**S2.** Checklist for reporting results of internet e-surveys. (CHERRIES) for Phase 1.

| ***Checklist Item*** | ***Explanation*** |
| --- | --- |
| Describe survey design | *Describe target population, sample frame. Is the sample a convenience sample? (In “open” surveys this is most likely.)*  As described in Methods, we took a convenience sample of volunteers. Eligibility criteria included being above the age of 50, located within the United States, able to read English, and self-identifying as having a chronic health condition. |
| IRB approval | *Mention whether the study has been approved by an IRB.*  As described in Methods, all study parts received IRB approval. |
| Informed consent | *Describe the informed consent process. Where were the participants told the length of time of the survey, which data were stored and where and for how long, who the investigator was, and the purpose of the study?*  Participants completed an IRB-approved consent form detailing the study’s purpose, time commitment, incentives, investigator identities, and data storage procedures. |
| Data protection | *If any personal information was collected or stored, describe what mechanisms were used to protect unauthorized access.*  Participant responses were kept within a secure REDCap database. A de-identified dataset was downloaded for further analysis. |
| Development and testing | *State how the survey was developed, including whether the usability and technical functionality of the electronic questionnaire had been tested before fielding the questionnaire.*  The survey was built on the REDCap platform and tested independently by two researchers before launch. |
| Open survey versus closed survey | *An “open survey” is a survey open for each visitor of a site, while a closed survey is only open to a sample which the investigator knows (password-protected survey).*  The survey was closed to ResearchMatch volunteers who met eligibility criteria. |
| Contact mode | *Indicate whether or not the initial contact with the potential participants was made on the Internet. (Investigators may also send out questionnaires by mail and allow for Web-based data entry.)*  Yes, participants received a notice about the study through email from ResearchMatch. |
| Advertising the survey | *How/where was the survey announced or advertised? Some examples are offline media (newspapers), or online (mailing lists – If yes, which ones?) or banner ads (Where were these banner ads posted and what did they look like?). It is important to know the wording of the announcement as it will heavily influence who chooses to participate. Ideally the survey announcement should be published as an appendix.*  Participants received an email from ResearchMatch if they met eligibility criteria. |
| Web/E-mail | *State the type of e-survey (eg, one posted on a Web site, or one sent out through e-mail). If it is an e-mail survey, were the responses entered manually into a database, or was there an automatic method for capturing responses?*  The survey was accessible by email to ResearchMatch volunteers. REDCap automatically captures responses in its own secure databases. |
| Context | *Describe the Web site (for mailing list/newsgroup) in which the survey was posted. What is the Web site about, who is visiting it, what are visitors normally looking for? Discuss to what degree the content of the Web site could pre-select the sample or influence the results. For example, a survey about vaccination on a anti-immunization Web site will have different results from a Web survey conducted on a government Web site*  ResearchMatch is an NIH-led participant database. Generally, we expect that participants are more likely to have a health condition and have higher education levels to willingly volunteer to participate in research, which was reflected in our sample. |
| Mandatory/voluntary | *Was it a mandatory survey to be filled in by every visitor who wanted to enter the Web site, or was it a voluntary survey?*  Participants volunteered to complete surveys via ResearchMatch. |
| Incentives | *Were any incentives offered (eg, monetary, prizes, or non-monetary incentives such as an offer to provide the survey results)?*  Yes, participants were entered into a lottery for 1 of 20 gift cards worth US $20. |
| Time/Date | *In what timeframe were the data collected?*  Data were collected in August 2022. |
| Randomization of items or questionnaires | *To prevent biases items can be randomized or alternated.*  Questionnaires and items were presented in a static order. |
| Adaptive questioning | *Use adaptive questioning (certain items, or only conditionally displayed based on responses to other items) to reduce number and complexity of the questions.*  Adaptive questions were not available for the measures we used. |
| Number of Items | *What was the number of questionnaire items per page? The number of items is an important factor for the completion rate.*  Some pages had only a single item; some had up to 18 items. |
| Number of screens (pages) | *Over how many pages was the questionnaire distributed? The number of items is an important factor for the completion rate.*  The total number of items was 49 across all questionnaires and demographics. |
| Completeness check | *It is technically possible to do consistency or completeness checks before the questionnaire is submitted. Was this done, and if “yes”, how (usually JAVAScript)? An alternative is to check for completeness after the questionnaire has been submitted (and highlight mandatory items). If this has been done, it should be reported. All items should provide a non-response option such as “not applicable” or “rather not say”, and selection of one response option should be enforced.*  Responses were checked for completeness after they had been submitted. Participants were excluded from individual analyses if they were missing a specific question answer. Participants were only wholly excluded if they never reached the end page of the survey. |
| Review step | *State whether respondents were able to review and change their answers (eg, through a Back button or a Review step which displays a summary of the responses and asks the respondents if they are correct).*  Participants could use a Back button at every point of the survey. |
| Unique site visitor | *If you provide view rates or participation rates, you need to define how you determined a unique visitor. There are different techniques available, based on IP addresses or cookies or both.*  Not applicable. |
| View rate (Ratio of unique survey visitors/unique site visitors) | *Requires counting unique visitors to the first page of the survey, divided by the number of unique site visitors (not page views!). It is not unusual to have view rates of less than 0.1 % if the survey is voluntary.*  Not applicable. |
| Participation rate (Ratio of unique visitors who agreed to participate/unique first survey page visitors) | *Count the unique number of people who filled in the first survey page (or agreed to participate, for example by checking a checkbox), divided by visitors who visit the first page of the survey (or the informed consents page, if present). This can also be called “recruitment” rate.*  As described in methods, 378 unique people began the survey. |
| Completion rate (Ratio of users who finished the survey/users who agreed to participate) | *The number of people submitting the last questionnaire page, divided by the number of people who agreed to participate (or submitted the first survey page). This is only relevant if there is a separate “informed consent” page or if the survey goes over several pages. This is a measure for attrition. Note that “completion” can involve leaving questionnaire items blank. This is not a measure for how completely questionnaires were filled in. (If you need a measure for this, use the word “completeness rate”.)*  57% of people who began the survey filled it out to completion. |
| Cookies used | *Indicate whether cookies were used to assign a unique user identifier to each client computer. If so, mention the page on which the cookie was set and read, and how long the cookie was valid. Were duplicate entries avoided by preventing users access to the survey twice; or were duplicate database entries having the same user ID eliminated before analysis? In the latter case, which entries were kept for analysis (eg, the first entry or the most recent)?*  Cookie settings were kept at the REDCap default. No duplicate responses were found based on identifiable participant information (e.g., email addresses submitted for payment). |
| IP check | *Indicate whether the IP address of the client computer was used to identify potential duplicate entries from the same user. If so, mention the period of time for which no two entries from the same IP address were allowed (eg, 24 hours). Were duplicate entries avoided by preventing users with the same IP address access to the survey twice; or were duplicate database entries having the same IP address within a given period of time eliminated before analysis? If the latter, which entries were kept for analysis (eg, the first entry or the most recent)?*  IP address was not collected to support participant anonymity. Based on other identifiable information (e.g., email address for payment), no duplicate responses were submitted. |
| Log file analysis | *Indicate whether other techniques to analyze the log file for identification of multiple entries were used. If so, please describe.*  No identifiable information (e.g., email address submitted for payment) indicated duplicate responses. |
| Registration | *In “closed” (non-open) surveys, users need to login first and it is easier to prevent duplicate entries from the same user. Describe how this was done. For example, was the survey never displayed a second time once the user had filled it in, or was the username stored together with the survey results and later eliminated? If the latter, which entries were kept for analysis (eg, the first entry or the most recent)?*  Participants could only begin the survey once via the ResearchMatch email sent to them. |
| Handling of incomplete questionnaires | *Were only completed questionnaires analyzed? Were questionnaires which terminated early (where, for example, users did not go through all questionnaire pages) also analyzed?*  As described in Methods, 124 participants did not read the end of the study and were thus wholly excluded. |
| Questionnaires submitted with an atypical timestamp | *Some investigators may measure the time people needed to fill in a questionnaire and exclude questionnaires that were submitted too soon. Specify the timeframe that was used as a cut-off point, and describe how this point was determined.*  Responses were not analyzed for completion time. |
| Statistical correction | *Indicate whether any methods such as weighting of items or propensity scores have been used to adjust for the non-representative sample; if so, please describe the methods.*  Responses were not weighted for analysis. We did conduct some analyses to see if subgroups (sex, age) differed significantly in their response patterns. |

This checklist has been modified from Eysenbach G. Improving the quality of Web surveys: the Checklist for Reporting Results of Internet E-Surveys (CHERRIES). J Med Internet Res. 2004 Sep 29;6(3):e34 [erratum in J Med Internet Res. 2012; 14(1): e8.]. Article available at [https://www.jmir.org/2004/3/e34](https://www.jmir.org/2004/3/e34/)/; erratum available <https://www.jmir.org/2012/1/e8/>. Copyright ©Gunther Eysenbach. Originally published in the [Journal of Medical Internet](http://www.jmir.org) Research, 29.9.2004 and 04.01.2012.

**Table S1A**. Modified version of the Media and Technology Usage and Attitude scale used in Phase 1.

| Item | Original Item, Modified from Original, or New? | Mean (SD) | Welch’s T-Test Results By Cancer Diagnosis | Welch’s T-Test Results By Sex | OLS Regression Results By Age |  |  |
| --- | --- | --- | --- | --- | --- | --- | --- |
| *Mobile Phone Items* | | | | |  |  |  |
| Use your mobile phone during working hours. | Modified | 6.16 (3.22) | n.s. | n.s. | n.s. |  |  |
| Use your mobile phone during non-working hours. | New | 8.09 (1.58) | n.s. | n.s. | n.s. |  |  |
| Send and receive text messages on a mobile phone. | Original | 7.47 (1.88) | n.s. | n.s. | β=-.05, P=.002 |  |  |
| Make and receive mobile phone calls. | Original | 6.81 (2.06) | n.s. | n.s. | n.s. |  |  |
| Read e-mail on a mobile phone. | Original | 6.92 (2.51) | n.s. | n.s. | β=-.07, P=.005 |  |  |
| Send e-mail on a mobile phone. | Modified | 5.86 (2.73) | n.s. | n.s. | n.s. |  |  |
| Browse the web on a mobile phone (for any purpose). | Modified | 6.73 (2.44) | n.s. | n.s. | β=-.08, P<.001 |  |  |
| Browse the web on a mobile phone for health information or health-related activities. | New | 4.50 (2.55) | n.s. | n.s. | n.s. |  |  |
| Use apps (for any purpose) on a mobile phone. | Original | 7.18 (2.38) | n.s. | n.s. | β=-.06, P=.02 |  |  |
| Use apps on a mobile phone for health information or health-related activities. | New | 4.51 (2.65) | n.s. | n.s. | n.s. |  |  |
| Play games on a mobile phone. | Modified | 4.40 (3.20) | n.s. | t(105.2)=3.41, P=.01 | n.s. |  |  |
| *Laptop/Desktop Computer Items* | | | | |  |  |  |
| Use your laptop or desktop computer during working hours. | New | 5.89 (3.63) | n.s. | n.s. | n.s. |  |  |
| Use your laptop or desktop computer during non-working hours. | New | 6.44 (2.45) | n.s. | n.s. | n.s. |  |  |
| Read email on a laptop or desktop computer. | New | 6.34 (2.66) | n.s. | n.s. | n.s. |  |  |
| Send email on a laptop or desktop computer. | New | 6.02 (2.72) | n.s. | n.s. | n.s. |  |  |
| Browse the web on a laptop or desktop computer (for any purpose). | New | 6.37 (2.75) | n.s. | t(110.5)=-3.26, P=.001 | n.s. |  |  |
| Browse the web on a laptop or desktop computer for health information or health-related activities. | New | 4.19 (2.34) | n.s. | n.s. | n.s. |  |  |
| Play games on a laptop or desktop computer. | Modified | 2.89 (2.79) | n.s. | n.s. | n.s. |  |  |

*Note*. *Original* items appeared as-is in the original Media and Technology Usage and Attitude scale. *Modified* items have the same meaning to an original scale item, but with adjusted wording. *New* items were created for the purposes of this survey. All items were rated on a scale of 1 (Never) to 10 (All the time). P values are adjusted with a Bonferroni correction for multiple comparisons and are only reported for P<.05.

**Table S1B**. Comparing matched items for mobile phone versus computers from Phase 1.

| Item | t-value | p-value |
| --- | --- | --- |
| Use your [mobile phone/laptop or desktop computer] during working hours. | 1.07 | .57 |
| Use your [**mobile phone**/laptop or desktop computer] during non-working hours. | 8.48 | <.001* |
| Read email on a [mobile phone/laptop or desktop computer.] | 2.32 | .11 |
| Send email on a [mobile phone/laptop or desktop computer.] | -0.56 | .58 |
| Browse the web on a [mobile phone/laptop or desktop computer] (for any purpose). | 1.44 | .46 |
| Browse the web on a [mobile phone/laptop or desktop computer] for health information or health-related activities. | 1.58 | .46 |
| Play games on a [**mobile phone**/laptop or desktop computer.] | 5.89 | <.001* |

P-values are adjusted with a Bonferroni correction for multiple comparisons.

**Table S2**. Abbreviated list of gameful design elements used in Phase 1.

| Item | Mean (SD) | By cancer diagnosis | By sex | By age |
| --- | --- | --- | --- | --- |
| *Learning*: Being invited to learn new skills that may be useful inside the system or in real life. | 4.3 (0.75) | n.s. | n.s. | n.s. |
| *Points*: Receiving points or experience for completing specific tasks. Points may be used to redeem rewards or towards progression. | 4.0 (0.98) | n.s. | n.s. | Higher age meant lower ratings, t(212)=-3.6, P=.007 |
| *Challenges*: Tackling difficult tasks to test my knowledge or skills. | 3.99 (0.88) | n.s. | n.s. | n.s. |
| *Quests*: Being invited to complete specific tasks to achieve meaningful goals. | 3.98 (0.84) | n.s. | n.s. | n.s. |
| *Meaning or Purpose*: Understanding that my effort will fulfill a meaningful goal (real or virtual) or feeling that I am part of something greater than myself. | 3.98 (0.90) | n.s. | n.s. | n.s. |
| *Meaningful choices*: I can choose between different ways of completing tasks or different rewards and the choices can lead to different results. | 3.96 (0.76) | n.s. | n.s. | n.s. |
| *Levels or Progression*: Being informed how much I have progressed in the system and how much I can still go to reach the top. | 3.90 (0.87) | n.s. | n.s. | n.s. |
| *Tutorials*: Getting used to the system with a tutorial or introduction on how everything works (also known as onboarding). | 3.88 (0.84) | n.s. | n.s. | n.s. |
| *Exploratory tasks*: Being free to explore the application or game and discover new ways of interacting with it. | 3.81 (0.94) | n.s. | n.s. | n.s. |
| *Customization*: Customizing my experience and how I present myself to others. | 3.73 (0.93) | n.s. | n.s. | n.s. |
| *Narrative or Story*: The system tells a story and lets me be part of the story through my actions and decisions. | 3.48 (0.98) | n.s. | n.s. | n.s. |
| *Badges or Achievements*: Receiving recognition for accomplishing meaningful goals inside the application or game. | 3.41 (1.00) | n.s. | n.s. | n.s. |
| *Collection*: Completing collections of items or achievements with special meaning in the application or game. | 3.37 (0.95) | n.s. | n.s. | n.s. |
| *Theme*: The system is described by means of a real or fictional central theme. | 3.21 (0.86) | n.s. | n.s. | n.s. |
| *Certificates*: Receiving certificates for completing special challenges or achievements. | 3.12 (1.06) | n.s. | n.s. | n.s. |
| *Avatar*: Being represented in the game or system by a customizable digital character (avatar). | 3.13 (1.00) | n.s. | n.s. | Higher age meant lower ratings, t(214)=-3.2, P=.03 |
| *Virtual World*: The game happens in a virtual world where players inhabit and interact. | 2.98 (1.12) | n.s. | n.s. | n.s. |
| *Leaderboards*: Comparing myself to others and show my status to others. | 2.78 (1.05) | n.s. | n.s. | n.s. |

*Note*. Participants rated each element on a five-point scale from 1 (Very unappealing) to 5 (Very appealing) depending on how they would appear in a mobile app to help manage health. Here, they are ordered from most to least appealing.

**S3:** Phase 2 wireframe interview guide.

**Introduction**

Hello, thank you for taking the time to talk with us today about the Cancer Symptom Check-in App. My name is [say name]. We are creating an app for patients with cancer to use for reporting and tracking their symptoms. Right now, we have produced a general concept for how we think the app might look, feel, and work. This was based on an initial survey of patients’ preferences.

Before we begin to develop a fully functioning app, we are looking for more in-depth feedback from a handful of patients like yourself. We want to make sure that what we create is appealing and useful to patients. Your honest opinions and advice are critically important to this goal and we very much appreciate any and all feedback you can provide.

Our interview today will last from 20 to 30 minutes. During the interview, we will show you some sample website screens and features and ask you open-ended questions for feedback. You will be compensated in the form of a $100 Visa Gift Card.

Do you have any questions before we begin?

Please let us know at any time during the interview if any questions arise or if you would like to stop the interview.

**Concept 1**

Our initial survey suggested that some users may find it engaging to report and track their symptoms using an app that has the look and feel of a computer or video game. Survey participants provided their preferences for different game design elements. Based on our results, one idea we brainstormed for the app is that users will progress through a virtual journey around the world while they answer questions about their symptoms on a routine basis (e.g., daily, weekly). Each symptom assessment will be completed in a different location on the virtual map. In order to progress to a new location on the map, users will need to complete their symptom questionnaire.

Now I’m going to show you a few of the sample screens we’ve come up with. We’ll click through several screens and explore the app. If you have any immediate thoughts or feedback we’d love to hear it. Then at the end we’ll ask several specific questions as well.

1. *Show home screen*: You’ll have several different options on the home screen. For now I’d like to focus on the main app function, the Daily Check-In, and walk you through it.
2. *Walk through daily check-in process*: First, you’ll select your destination. You can resume travel at unlocked destinations… [Walk through question response process that is interspersed with travel facts. Stop at the finished itinerary screen, before viewing results.]

STOP at “Finish” button and ask:

Major question: What are your thoughts on the concept of having the app guide you through a virtual journey around the world?

*Probes*:

1. Is the feature of being able to travel to a new location within the virtual map motivating? Why or why not?
2. How do you feel about the “fun fact” feature between questions? Would you use it or turn it off?
3. We’re considering a feature that rewards users with “frequent flyer points” after completing a questionnaire. Do you like the idea of frequent flyer points that you could redeem to travel to a new location? Why or why not?
4. What about a feature that rewards users by collecting badges from your travels to new locations, such as stamps on a virtual passport book?
5. What about world tour “challenges” or races, to travel to a certain number of destinations in a certain amount of time?

**Concept 2**

To help with monitoring and managing symptoms, we’re thinking that the app could provide users with immediate feedback about whether each symptom assessment was low, average, or high. Users would also be able to view a personalized dashboard with a graph displaying their previous symptom scores over time.

Here are some sample website screens:

1. *Show feedback screen* – explain score ranges; color coding of PROMIS T-scores for normal, mild, moderate, and severe score ranges; and graphs of trends over time.
2. *Show tips screen* – describe tips as daily pop-ups that give small pieces of information about app function reminders and/or symptom management advice.

Major question: Would you find symptom feedback and tracking over time helpful? Why or why not?

*Probes*:

1. If you were to score high on a symptom, what sort of support from the app would you want to understand your result or help manage the symptom?
2. Do you like the idea of being able to choose which symptom or symptoms you would like the app to focus on?
3. Do you find the graphs with your scores over time useful? Why or why not?

**General Questions**

Now we’d like to ask you about your impressions overall.

1. Could you imagine yourself using an app with the features we’ve discussed today? Why or why not? How often would you use it?
2. Can you imagine other people with cancer using this app? Why or why not?
3. How do you currently monitor symptoms? Would the app be helpful for symptom monitoring?
4. What feature or features were most appealing to you?
5. What feature or features were least appealing to you?
6. Is there anything you would change or improve about the app?
   a. About the travel concept?
   b. About symptom monitoring?

**Closeout**

We would like to ask you three questions about your demographics. This is to provide broad-level context about our website development process. This information will not be attached to your comments from the interview.

- What is your age?
- What is your sex?
- What is your cancer diagnosis?

**S4:** Phase 3 clinician interview guide.

**Introduction**

Hello, thank you for taking the time to talk with us today about the Cancer Symptom Tracking App called AthenaCompanion. My name is [Insert name]. We have developed an app and are conducting a study to improve engagement among cancer survivors.

Before we begin refining the app, we are looking for more in-depth feedback from a handful of providers like yourself. We want to ensure that what we create is appealing and useful. Your honest opinions and advice are critically important to this goal, and we very much appreciate any feedback you can provide.

Our interview today will last from 20 to 30 minutes. During the interview, we will show you the app and how it works. We will also show you 3 examples of reports generated by the app that could be reviewed by providers. We will ask your thoughts and opinions on how you interpret the reports, and how we can improve the reports. You will be compensated through a $40 Gift Card.

**App Walkthrough**

Demonstrate the main features of the app live to the clinician.

- Home screen with multiple tiles for specific features
- Walk through a check-in: selecting a destination, answering each PROMIS question, travel facts interspersed with questions, final screen showing scores, symptom trend display over time, generate a report and email it button.

*Questions*:

- What are your thoughts on the app?
- As you saw, the reports can be emailed to anyone the patient chooses, including themselves or their provider. How would you want to see or be presented with reports? On the patient’s phone at an appointment? Via email from the patient? Attached to a MyChart message?

**Report Walkthrough**

We have generated 3 reports from fictional patients whose symptoms over time are different. When I show you each report, I would like you to think aloud as you are viewing the report: tell me what you are looking at, how you think about what it means, and similar.

[Start with any of the 3 reports; start with different reports for each subsequent interview. (The Irma report is a patient who is relatively stable; the Taylor report is a patient who has slowly declined in several symptoms; the Greg report is a patient who sharply declined in one symptom only.)]

*Questions:*

- Could you please interpret this report?
- What would you ask the patient about the report?
- What action would you take, if any, after reading this report?
- What data on the report is useful? What is not useful?

After doing at least 2 reports, move on to the following questions.

- For the scores you’ve seen so far like Fatigue and Depression, higher scores are worse functioning and lower scores are better functioning. But, some symptoms in the PROMIS family are scored on an *opposite* metric: a high Physical Function score means that the patient is functioning very well. How would you want this displayed and communicated on a report? Separate pages? Same graph, but scores reversed?
- How far back would you want to see patient data? One month? Six months? The last X reports? High/low scores of all time?

**S5:** Phase 3 patient usability test guide.

**Introduction**

Hello, thank you for taking the time to talk with us today about the Cancer Symptom Tracking App called ***AthenaCompanion***. My name is [Insert name], and this is [insert name(s)]. We have developed an app and are conducting a study to improve engagement among cancer survivors.

Before we begin refining the app, we are gathering feedback from a handful of survivors like yourself. We want to ensure that what we create is appealing and helpful to patients. Your honest opinions and advice are critically important to this goal, and we very much appreciate any feedback you can provide.

Our interview today will last about 60 minutes. During the interview, we will ask you to complete a usability session, followed by an interview to discuss your impressions. You will be compensated through a $40 Gift Card. One week from today, we will send you a follow-up survey. If you complete that survey, you will receive an additional $10 Visa gift card.

Do you have any questions before we begin?

Please let us know anytime during the interview if any questions arise or if you want to stop. First, we would like to ask some questions about you and tracking symptoms during treatment.

What is your age? __________________

What is your sex? __________________

What is your cancer diagnosis? __________________

**Usability Test**

Now, we will ask you to complete some tasks in the app while thinking aloud.

What is thinking aloud?: As part of this usability test, we’d like you to use the app and share your thoughts out loud as you interact with it. This is called the “think aloud” method. While exploring the app, we want you to say whatever comes to mind — things you notice, decisions you’re making, questions you have, or anything you find confusing.

There are no right or wrong things to say. For example, you might mention:

- What you’re looking for or trying to do.
- Why you’re clicking on something or hesitating to make a choice.
- Anything you find helpful or challenging.

Your feedback is essential because it will help us understand how the app works for you and improve it for others.

If you forget to think aloud, don’t worry! We might remind you to share your thoughts. Just focus on being yourself and using the app naturally.

Now, if it is ok with you, I will ask you to **please share your screen** so that if you get stuck, I can help you.

[Note: If stuck, after ~1-2 minutes, move on to the next task.]

***Task #1***

1. Look for your registration email that was just sent to you. The email contains a link to set your new password and access the app.

2. Follow the link to set your password.

3. Once you’ve set your password, log in to the app.

4. When the setup screen appears, you’ve completed this task.

[Facilitator Note: If the participant is not thinking aloud, prompt them to do so. Re-read instructions if requested. Encourage them to attempt the task independently before offering assistance.]

***Task #2***

1. From the setup screen, configure the app to track three symptoms: depression, fatigue, and physical function.

2. At this point, this task is complete.

***Task #3***

1. Set a text reminder every Monday at 1:00 PM in your time zone.

2. Save your settings.

3. Once saved, you will be directed to the home screen. At this point, this task is complete.

***Task #4***

1. Navigate to your email and watch the introduction tutorial video.

2. Navigate back to the app and look at the content on the home screen.

3. Without clicking on anything, interpret what you see.

4. Once you’ve reviewed the content, the task is done.

***Task #5***

1. From the home screen, use the app to track the three symptoms you selected during setup.

2. When you’ve finished reporting your symptoms, this task is done.

***Task #6 [Check-In Report]***

1. After reporting your symptoms, navigate to the next page.

2. Watch the video at the top of the page

3. Find and tell us your physical function score.

4. Once this is done, the task is complete.

***Task #7 [Check-In Report]***

1. Identify which symptoms (if any) suggest speaking to a provider.

2. Once you’ve done this, the task is complete.

***Task #8 [Symptom Trends]***

1. From here (check in report), find the page displaying symptom trends.

2. Watch the video at the top of the page.

3. Interpret the information on this page.

4. Once you’ve done this, the task is complete.

***Task #9 [PDF Report]***

1. From here (symptom trends page), locate and download your symptom report.

2. Open the report.

3. Find and state the scores for each of the three symptoms you reported.

4. Once you’ve completed this, the task is done.

***Task #10 [View Past Reports]***

1. Navigate back to the home screen.

2. Find the report from today’s check-in, which includes the green-yellow-red scale.

3. Add a note: “I went for a long walk today.”

4. Save the note, once you’ve done this, the task is complete.

***Task #11 [Symptom Trends]***

1. From the home screen, navigate to the page with the graph showing symptom trends.

2. Locate and tell us your physical function score on the graph.

3. Once you’ve done this, the task is complete.

***Task #12 [Settings]***

1. Navigate to the settings page from the home screen.

2. Add a new symptom of your choice.

3. Turn off SMS notifications.

4. Once completed, the task is done.

***Task #13 [PDF Report]***

1. From the home screen, locate and open the PDF report.

2. Find the note you left on the check-in page.

3. Once you’ve done this, the task is complete.

***Task #14 [Help]***

1. From the home page, find the tutorial video that explains the app’s features.

2. Once you’ve located the video, the task is complete.

**Post Usability Test Interview Questions**

- What are your thoughts on this app?
- Can you describe the reasons for using or not using this app to track your symptoms?
- How might you see yourself using this app, and what would encourage you to use it regularly? How could it be improved?
- Which app feature(s) did you like the most? What about the least?
- Do you have any suggestions on how this app could be more useful to you?

Interviewer shares their screen and PowerPoint. Navigate to the *View Past Reports* page.

- What are your thoughts on this page?
- Probe: When an image with distinct scores at each category is available, ask for interpretation. If you were to score Normal, Mild, Moderate, and Severe on a symptom, what sort of support from the app would you want to understand your result?

Navigate to *Symptom Trends*.

- What are your thoughts about these graphs?
- What would you like to see?

Navigate to the *PDF Report*.

- What are your thoughts on the PDF reports?
- What would you like to see?
- Would you ever consider sharing this with your medical team? Why or why not?

Navigate to the *Passport*.

- What are your thoughts on the passport feature where you travel to a new location to complete your symptom check-in?
- What are your thoughts on the “fun facts” between the questions of each symptom?
- Would this feature prevent you from reporting your symptoms?
  **Table S3.** Themes from Phase 2 patient interviews.

| **Theme** | **Summary of Findings** |
| --- | --- |
| Interest in symptom tracking and visualization | 7/7 (100%) participants found color-coded symptom severity on individual reports and longitudinal graphs intuitive and useful for self-monitoring and communicating with clinicians; 6/7 (86%) cited this as their favorite feature. |
| Desired support for high symptom burden | 6/7 (85%) of participants expressed interest in in-app education and lifestyle guidance if scoring high; some participants (4/7, 57%) wanted an in-app option to contact their provider. |
| Reactions to gamified travel features | Positive-to-mixed; 2/7 (29%) listed this as their least favorite feature. |
| Preferences for gamification elements | When given examples of potential gamification features (points, stamps, streaks, timed challenges), passport stamps were favored while streaks and timed challenges had mixed-to-negative reactions. |
| Intention to use app | 6/7 (86%) of participants said they would use the app as it was at the time of the interview if it were publicly available. |
